# Biology of Saccular Cerebral Aneurysms: A Review of Current Understanding and Future Directions

**DOI:** 10.3389/fsurg.2016.00043

**Published:** 2016-07-25

**Authors:** Vernard S. Fennell, M. Yashar S. Kalani, Gursant Atwal, Nikolay L. Martirosyan, Robert F. Spetzler

**Affiliations:** ^1^Department of Neurosurgery, Barrow Neurological Institute, St. Joseph’s Hospital and Medical Center, Phoenix, AZ, USA

**Keywords:** biology, inflammation, intracranial aneurysm

## Abstract

Understanding the biology of intracranial aneurysms is a clinical quandary. How these aneurysms form, progress, and rupture is poorly understood. Evidence indicates that well-established risk factors play a critical role, along with immunologic factors, in their development and clinical outcomes. Much of the expanding knowledge of the inception, progression, and rupture of intracranial aneurysms implicates inflammation as a critical mediator of aneurysm pathogenesis. Thus, therapeutic targets exploiting this arm of aneurysm pathogenesis have been implemented, often with promising outcomes.

## Introduction

Intracranial aneurysms are common abnormalities of the brain (1–30). The reported prevalence was 3.2% in a homogeneous Finnish population and up to 5% in others (31, 32). The overall risk of rupture is about 1% (33, 34). At 40–65%, the overall lethality of subarachnoid hemorrhage (SAH) resulting from cerebral aneurysm rupture is significant (31, 35, 36). Thus, SAH remains a challenging clinical issue (31, 32, 37–43). Of patients who survive the initial ictus, ≤50% face significant morbidity (31, 38, 40, 44, 45).

The true natural history of cerebral aneurysms is incompletely understood. Types of cerebral aneurysms include giant, fusiform, and saccular. In this review, we focus on saccular aneurysms. Although much of the aneurysm biology remains unknown, a growing body of literature addresses their formation, progression, and rupture.

## Clinical Risk Factors

Risk factors for intracranial aneurysms include the epidemiological risk factors of female sex, smoking, hypertension, and family history, which is the strongest indicator of rupture among non-modifiable risk factors. Compared to the general population, first-degree relatives of persons with intracranial aneurysms or previous SAH have a risk 3–7 times higher and tend to have ruptured aneurysms at younger ages than those with sporadic aneurysms (37, 38, 40, 42, 46–48). In a cohort of 142 patients with 181 unruptured aneurysms followed from the 1950s until 1997–1998 for death or SAH, the annual incidence of hemorrhage was 1.3% (36). Cumulative rates of bleeding were 11% at 10 years, 23% at 20 years, and 30% at 30 years. Associated risk factors were aneurysm diameter and age. Smoking was an independent covariate related to rupture risk.

## Anatomical and Circulatory Factors

Aneurysms develop at branch points of high intravascular turbulence and abnormal vessel wall shear stress. They arise in areas with complex arterial vascular geometry, particularly bifurcations and curvatures that contribute to increases in wall shear stress. Although formation is linked to diffuse genetic/familial, environmental, and immunologic risk factors, saccular aneurysms seldom occur in random locations (31, 43). They tend to arise in sites similar to where giant and fusiform aneurysms form, with comparable and predictable geometric and anatomical properties. Vascular flow is turbulent or laminar. Turbulent flow has random variations in temporal and spatial components, with inconsistent predictability (43). Laminar flow typically occurs in large, straight vessels and is synonymous with normal physiological conditions but can be more complex or “disturbed,” occurring in areas of arterial bifurcations or poststenotic areas (49–52). These perturbations in flow often result in endothelial dysfunction, aiding aneurysm formation (31, 43). The endothelial response to wall shear stress appears to cause a cascade of gene signaling, morphological, and phenotypic changes that result in the initiation, progression, and rupture of intracranial aneurysms.

The locations of aneurysms are relatively consistent, with most cerebral aneurysms in the circle of Willis (43). However, considerable anatomical variability results from population-level differences in the individual geometry of the circle of Willis. Only 40% of people have a characteristic “complete” circle of Willis (43, 53). Unlike most large extracranial arteries, the bifurcation apex in cerebral vessels does not have consistent histologic media. Furthermore, the cerebral bifurcation apex has significantly less structural support from perivascular tissue (43, 54). Hemodynamic data suggest that deviations from optimal geometric constructs predispose specific vessels to aneurysm formation.

Approximately 90% of cerebral aneurysms occur in the anterior circulation, commonly (30–35%) the anterior communicating artery complex, followed by the internal carotid artery (30%) and associated branches (posterior communicating, ophthalmic arteries). Lastly, 22% occur in the middle cerebral artery and 10% in the posterior circulation (basilar apex, superior cerebellar artery, posterior inferior cerebellar artery) (40). These locations correlate with the distribution of intracranial atherosclerosis and areas of suboptimal hemodynamic patterns (40, 43). Known anatomical differences in familial aneurysms also account for approximately 10% of SAHs (38). Familial aneurysms typically are multiple and occur in the middle cerebral artery.

## Aneurysm Formation and the Role of Inflammation

Numerous immunologic factors may influence the formation of intracranial aneurysms and their progression and rupture.

### Pathology

The pathophysiological underpinnings of a saccular cerebral aneurysm may lie in an atherosclerotic pathway. Animal modeling points to damage of the internal elastic lamina that may define early aneurysm formation and change (55–60). Further atherosclerotic changes within the aneurysm wall are also described (61, 62). Structural differences occur in both small and large saccular aneurysms. Small aneurysms have diffuse intimal thickening, with proliferating vascular smooth muscle cells (VSMCs) and a preponderance of macrophages and lymphocytes. Larger aneurysms have more advanced atherosclerotic changes, particularly with phenotypic changes in VSMCs, lipid-laden macrophages, and lymphocytic infiltration.

Our current understanding of atherosclerosis as a contributor to cerebral aneurysm formation and progression is rooted in efforts to define abdominal aortic aneurysms (63–66). Individuals with both cerebral and abdominal aneurysms share comorbid risk factors, such as smoking and arterial hypertension. Immunologic response and chronic inflammation are key pathogenic features of atherosclerosis (67–73). These immunologic responses suggest that inflammatory mediators could be linked to the formation, progression, and rupture of cerebral aneurysms (31).

### Vessel Wall Changes

Histologic changes in aneurysm formation include vessel wall damage as a precursor. Normal vessel walls are organized into distinct layers, while aneurysmal vessel walls have fewer distinct layers characterized by disintegration of the internal elastic lamina, progressive disorganization of the muscular media, intimal hyperplasia, and progressive irregularity of the luminal surface (74–79). Healthy cerebral vessels have a mix of collagen and connective tissue (type I, III, and IV), fibronectin, and laminin. Type I collagen exists mostly in adventitia and fibronectin in the media of normal vessels (80). However, vascular remodeling changes the vessel wall. Type I collagen increases and fibronectin is dispersed in the wall, while the levels of type III and IV collagen and laminin decrease (54).

Structural and pathological changes occur in the endothelium and VSMCs. Functioning vascular endothelium promotes vasodilation and is antiatherogenic; it also inhibits platelet adhesion and accumulation, VSMC proliferation and leukocyte adherence, and pro-inflammatory cascades. Recent evidence points to damage of the vascular endothelium as the inciting event, leading to the creation, inflammatory cascade, progression, and rupture of intracranial aneurysms (81–83). The key inciting event in endothelial injury may be hemodynamic stress (76).

Perturbations in the vascular endothelium appear constant in both experimental and human intracranial aneurysms (75, 77, 81, 84–89). Damage to the vascular endothelium incites morphologic and pathologic changes likely occurring in stages. The earliest changes (e.g., partial loss of endothelium) occur upon aneurysm formation and the latest (e.g., intimal swelling) upon progression. Initial morphologic and functional changes in the endothelium could be a response to shear stress. Endothelial cells become elongated and realign with directional blood flow. Changes also occur in the development of actin stress fibers that may alter endothelial cell density or migration (90, 91). Hemodynamic stress may alter acute and chronic inflammatory signaling pathways. Shear stress appears to activate mediating pathways of inflammation within endothelial cells [prostaglandin E(2)–E-prostanoid(2) (PGE(2)–EP(2))]. It also may amplify the chronic inflammatory pathway *via* nuclear factor-κB (92).

Changes in vessel walls are punctuated by changes in the vascular endothelium that occur in concert with phenotypic and morphologic changes in VSMCs supporting the media layer of the intracranial vasculature and providing structural support to vessel walls. Dynamic changes and eventual loss of the media layer contribute to aneurysm formation and rupture (80). Histologic evidence suggests that normally contractile VSMCs respond to environmental cues by undergoing phenotypic changes causing them to resemble a pro-inflammatory, pro-remodeling, and dedifferentiated phenotype (93–95). Normal differentiation of cerebral VSMCs includes high levels of largely contractile proteins comprising smooth muscle-myosin heavy chains, smooth muscle alpha-actin, and semicarbazide amine oxidase, which regulate VSMC differentiation (54, 96–105). An early morphologic finding was related to phenotypic changes in these proteins. The spindle-like VSMCs change into spider-like cells that migrated to and proliferated in the media, resulting in myointimal hyperplasia (99). These changes may be punctuated by the previously mentioned hemodynamic factors, macrophage and endothelial cell-derived mediators [tumor necrosis factor (TNF)-α, interleukin (IL)-β, nitric oxide, and growth factors], environmental factors, and genetic changes (54, 100, 102, 104). This punctuated VSMC transition results in proliferation of a pro-inflammatory phenotype of VSMCs. The pro-inflammatory phenotype is characterized by reduced levels of the contractile elements of VSMCs: smooth muscle-myosin heavy chains, smooth muscle alpha-actin, and semicarbazide-sensitive amine oxidase (54, 102). Further changes in the increase in transcription factors (Ets-1, p47phox, IL-6, monocyte chemoattractant protein-1, reactive oxygen species, matrix metalloproteinases, cathepsins), promoting inflammation, recruiting reactive oxygen species, and matrix remodeling, are identified as potentially promoting aneurysm progression (96, 98, 103, 106). Ultimately, these changes result in decreased expression of collagen biosynthesis and further loss of VSMCs, weakening the aneurysm wall and predisposing to aneurysm rupture (31).

### Specific Inflammatory Pathways

**Figure 1:**
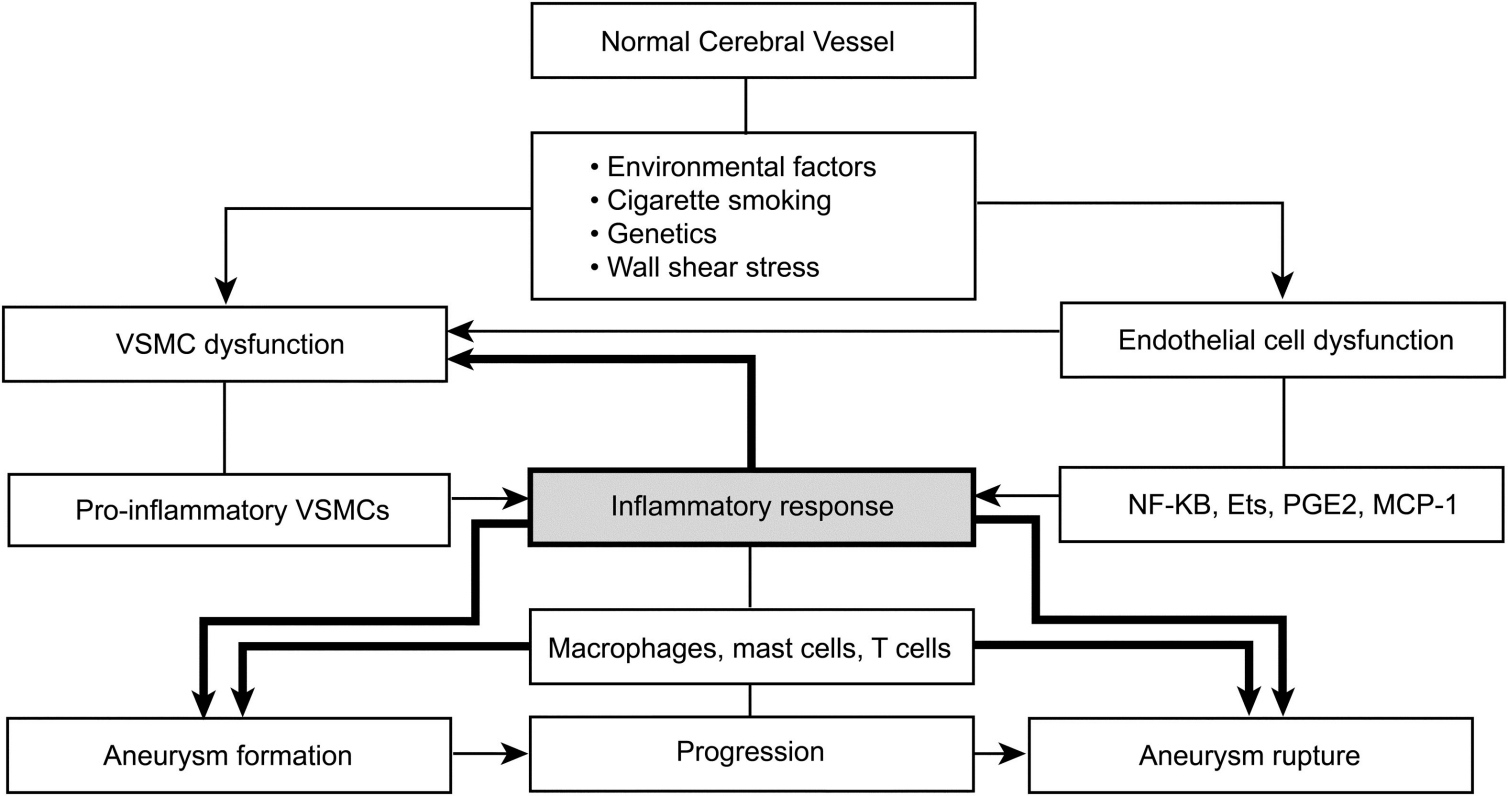
**Environmental factors and immunologic pathways and mediators involved in aneurysm formation**. Shading emphasizes the contribution of inflammation to the process of aneurysm formation. VSMC, vascular smooth muscle cell; NF-κB, nuclear factor-κB; Ets, E-twenty-six family transcription factors; PGE2, prostaglandin E2; MCP1, monocyte chemoattractant protein 1. *Used with permission from Barrow Neurological Institute, Phoenix, AZ, USA*.

The specific immunologic pathways and mediators involved in aneurysm formation remain partially understood. However, the immunologic effect can be divided into three areas linked to endothelial cells, VSMCs, and leukocytes. A common pathway for aneurysm formation is linked to certain leukocytes with distinct pathways of influence and known associated inflammatory mediators catalyzed by endothelial injury (31). The immunologic function is mediated by endothelial dysfunction, and the primary inflammatory mediators are NF-κB, Ets-1, MCP1, IL-1β, nitric oxide, angiotensin II, phosphodiesterase-4, and PGE(2)–EP(2) (Figure 1) (31). Dysfunctional major pathways of VSMCs include pro-inflammatory and pro-matrix remodeling, along with phenotypic modulation and associated apoptotic cell death. The major inflammatory mediators involved in VSMCs are IL-1β, p47phox, Ets-1, MCP1, angiotensin II, reactive oxygen species, matrix metalloproteinase, and cathepsins (31, 84, 107). Leukocytes, particularly mast cells and T-cells, influence aneurysm formation *via* a chronic inflammatory pathway associated with vessel wall remodeling and damage, with subsequent apoptotic cell death. Several inflammatory mediators are associated with leukocytes: TNF-α, IL-1β, IL-6, TLR4, Fas, nitric oxide, complement, IgG, IgM, basic fibroblast growth factor, TGF-α + β, vascular endothelial growth factor, reactive oxygen species, matrix metalloproteinases, and cathepsins (31, 108, 109). Understanding how these specific inflammatory mediators function opens the door to treatments targeting these major inflammatory pathways (31).

## Genetic Factors

Genetic factors contribute to the formation, progression, and rupture of intracranial aneurysms. Several studies have used microarray polymerase chain reaction to characterize the nature of these lesions. Despite further elucidating the gene expression profiles of these lesions, these studies have been limited by the significant variability of lesion types, stage of progression, location, and rupture status of lesions (31). The variability and small sample sizes in published gene expression studies impede generalizations to the intracranial aneurysm population.

Microarray data have yielded more than 500 differentially expressed genes in intracranial aneurysm tissue (31, 110). The two most significantly associated gene ontology terms identified were in antigen processing and immune response. Additional processing of aneurysm tissue revealed significant involvement, confirmed by real-time polymerase chain reaction, in integrin signaling, chemokine signaling, complement and coagulation cascades, nitric oxide signaling, and IL-10 signaling. These studies showed a convincing correlation of major histocompatibility complex II gene overexpression in aneurysm tissue that associated antigen-presenting cells, particularly macrophages and monocytes, with intracranial aneurysm formation (31). Gene analysis of a rodent aneurysm model has shown associations in pathways involved with proteinases, reactive oxygen species, chemokines, complement, adhesion molecules, and apoptotic pathways in both the intima and media of aneurysm walls (31, 111). These data also showed differential expression of endothelial cells and VSMCs, suggesting a different role in the process of aneurysm formation (31, 112).

Gene expression patterns were more recently studied in groups of ruptured and unruptured aneurysms (31, 113), with 686 upregulated and 740 downregulated genes identified in the ruptured cohort. Upregulated pathways were numerous, most notably in response to turbulent blood flow, chemotaxis, leukocyte migration, oxidative stress, extracellular matrix degradation, and vascular remodeling. Additionally, enriched genes encoding TLR, NF-κB, hypoxia-induced factor 1A, and Ets transcription factor-binding sites were identified. These findings suggest that, although both aneurysm groups have an immunologic pedigree, ruptured and unruptured aneurysms likely have different immunologic biology.

Known genetic conditions and familial relationships are also associated with higher rates of intracranial aneurysms. Autosomal polycystic kidney disease, Ehlers–Danlos syndrome, neurofibromatosis 1, and alpha1-antitrypsin deficiency are linked with aneurysm formation (31, 40). Thus, if there are definable immunologic pathways and common identifiable genomic markers, then multiple avenues may be available for preictal intervention.

## Future Directions and Treatments

Given our expanding understanding of the contribution of inflammatory factors to aneurysm formation, great efforts have been made in investigating non-interventional treatments. Much of the non-interventional therapeutic research to date has been conducted in animals, with the most promising data from studies on inhibiting the NF-κB pathway.

Multiple animal trials have sought to exploit the anti-inflammatory effect of statins. Statins can block different stages of the inflammatory reaction, decrease degeneration in the vessel, and slow intracranial aneurysm progression (3, 31, 85, 114). Unfortunately, other data indicate variable results with different doses of pravastatin (88). At lower doses (5 mg/kg/day), pravastatin reduced overall endothelial damage and inhibited aneurysm formation in rats (88). The reverse was noted at higher doses of pravastatin (25 and 50 mg/kg/day) and at lower doses of simvastatin (5 mg/kg/day), where there was enhancement of aneurysm growth, and with high-dose pravastatin, even induction of aneurysm rupture (31, 88). The adverse effects of statins were accompanied by increased apoptotic caspase-3 levels and TUNEL-positive cells. Positive but disparate results have also been found with a phosphodiesterase-4 inhibitor and several angiotensin II receptor blockers (3, 31, 81, 85, 115).

The most impressive animal data involve NF-κB inhibition in rats. A drastic decrease in inflammatory response and a 60% decrease in aneurysm incidence were found with NF-κB inhibition (31, 116). Whether the litany of animal data will have a translational impact remains to be seen. Retrospective data from the International Study of Unruptured Intracranial Aneurysms showed that patients who used aspirin three times weekly had a lower risk of aneurysm rupture versus those who did not use aspirin (117), perhaps because of the known anti-inflammatory effects of aspirin.

There are multiple, largely rat, studies of cathepsin inhibitors, MCP1 inhibitors, matrix metalloproteinase inhibitors, mast cell degranulation inhibitors, and free radical scavengers. These agents have diversely positive effects on factors, such as aneurysm incidence, size, media thickness, and internal elastic lamina score (2, 31, 97, 114, 118). The positive animal data continue to mount, prompting great hope it will translate into positive clinical therapies.

## Conclusion

There is still much to learn about aneurysm biology. Experimental animal data support inflammatory pathways as a key factor in aneurysm formation, progression, and rupture, but concrete non-surgical therapeutic targets remain elusive. Continued research and understanding of the biology and immunology of aneurysms have been pivotal in broadening our current understanding and will play an important role as we continue to improve the treatment of this pathology.

## Author Contributions

All authors listed have made substantial, direct, and intellectual contributions to the work and approved it for publication.

## Conflict of Interest Statement

The authors declare that the research was conducted in the absence of any commercial or financial relationships that could be construed as a potential conflict of interest.

## Abbreviations

IL, interleukin; NF-κB, nuclear factor-κB; SAH, subarachnoid hemorrhage; TNF, tumor necrosis factor; VSMC, vascular smooth muscle cell.
